# The Origin and Fate of Chondrocytes: Cell Plasticity in Physiological Setting

**DOI:** 10.1007/s11914-023-00827-1

**Published:** 2023-10-14

**Authors:** Andrei S. Chagin, Tsz Long Chu

**Affiliations:** 1 Department of Internal Medicine and Clinical Nutrition, Institute of Medicine, Centre for Bone and Arthritis Research at the Sahlgrenska Academy, University of Gothenburg, Gothenburg, Sweden; 2 Department of Physiology and Pharmacology, Karolinska Institute, Stockholm, Sweden

**Keywords:** None, Growth plate, Chondrocytes, Osteoblasts, Dedifferentiation, Transdifferentiation

## Abstract

**Purpose of Review:**

Here, we discuss the origin of chondrocytes, their destiny, and their plasticity in relationship to bone growth, articulation, and formation of the trabeculae. We also consider these processes from a biological, clinical, and evolutionary perspective.

**Recent Findings:**

Chondrocytes, which provide the template for the formation of most bones, are responsible for skeletal growth and articulation during postnatal life. In recent years our understanding of the fate of these cells has changed dramatically. Current evidence indicates a paradoxical situation during skeletogenesis, with some cells of mesenchymal condensation differentiating directly into osteoblasts, whereas others of the same kind give rise to highly similar osteoblasts via a complex process of differentiation involving several chondrocyte intermediates. The situation becomes even more paradoxical during postnatal growth when stem cells in the growth plate produce differentiated, functional progenies, which thereafter presumably dedifferentiate into another type of stem cell.

**Summary:**

Such a remarkable transition from one cell type to another under postnatal physiological conditions provides a fascinating example of cellular plasticity that may have valuable clinical implications.

## Mesenchymal Condensation and Formation of the Growth Plate and Articular Cartilage

With the exception of the facial bones, which arise from the neural crest and Schwann cell precursors [1, 2], skeletal elements are mesodermal in origin and these, with particular focus on the appendicular skeleton, will be discussed here.

The classic view is that in all vertebrates, bone formation is either intramembranous or endochondral. The former process involves the differentiation of mesenchymal cells, generated from the mesoderm, directly into osteoprogenitors and subsequently into osteoblasts, whereas in the latter, mesenchymal cells first differentiate into chondrocytes, and these cells then form a cartilaginous template which grows via the proliferation and hypertrophy of the chondrocytes, with subsequent replacement of this template by the bone.

In humans, as well as all other mammals, formation of the flat bones of the skull and portions of the scapula and clavicle is intramembranous. However, most of the bones in adult humans develop via endochondral ossification. This process has been extensively characterized and reviewed elsewhere [3–6], and here, we present only a brief overview of the main steps, highlighting those of direct relevance to our major topics.

**Fig. 1:**
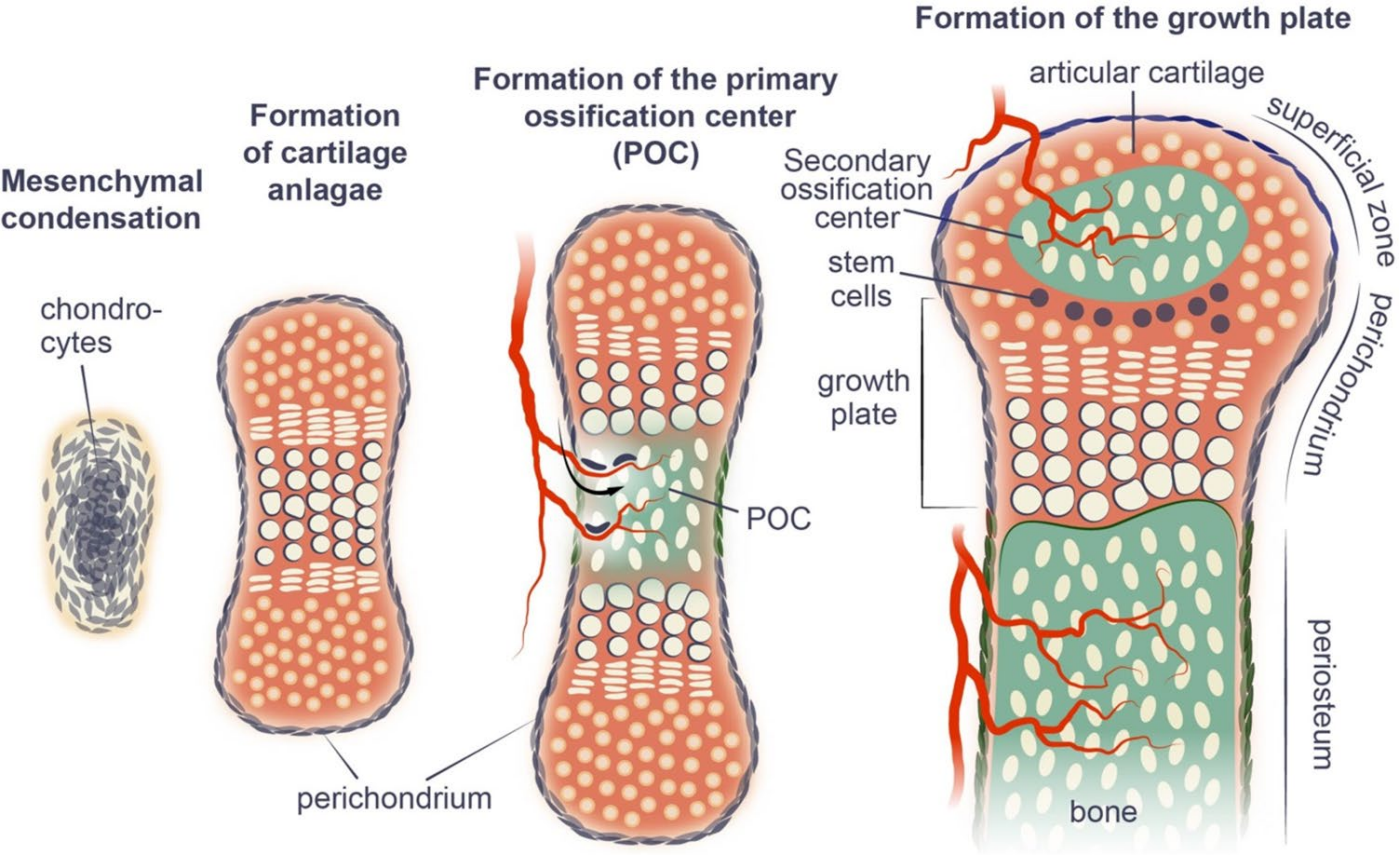
The different phases of endochondral bone formation

During mesenchymal condensation, cells differentiate into chondrocytes, which then proliferate and generate an extracellular matrix composed predominantly of collagens type II and XI, together with aggrecan, thereby forming the template referred to above (Fig. 1). Importantly, mesenchymal cells at the periphery behave differently, forming instead a sheet of tightly packed cells known as the perichondrium, which envelops the cartilage and grows with it (Fig. 1). The chondrocytes at the center of the cartilage template undergo hypertrophy, increasing as much as 20-fold in volume [7], probably due to hypoxia, since all cartilage is avascular [8].

At the same time, the extracellular matrix around hypertrophic chondrocytes becomes more enriched in collagen type X, and these chondrocytes begin to secrete several important signaling factors, including Indian hedgehog (IHH), vascular endothelial growth factor (VEGF), and metalloproteinases (MMPs) [9–11]. VEGF promotes invasion of the tissue by blood vessels, together with which osteoclasts (sometimes referred to as chondroclasts in connection with cartilage resorption), in combination with the MMPs secreted by hypertrophic chondrocytes, form inner bone marrow cavities. Traveling along blood vessels, cells of the perichondrium migrate into these cavities, where they give rise to osteoprogenitors and, subsequently, endosteal osteoblasts at the inner surfaces of the cavities [12].

This complex process results in the formation of the primary ossification center (POC) at the middle of the cartilaginous template (Fig. 1). The ends of the template on both sides of the POC, often referred to as cartilaginous epiphysis or epiphyseal growth plates, become polarized in the longitudinal direction and continue to expand, predominantly in this same direction. Thereafter, in the centers of these cartilaginous epiphyses, chondrocytes again undergo hypertrophy and blood vessels invade and, together with perichondrial cells [13], these structures form the secondary ossification center (SOC), which separates the cartilaginous epiphysis into the growth plate between the SOC and POC, on the one hand, and the articular cartilage at the topical side of the SOC, on the other [14] (Fig. 1). Functionally, the SOC provides mechanical protection for the underlying growth plate [15] and forms a stem cell niche for epiphyseal stem cells shown recently to reside at the top of the growth plate [16–18].

Thus, the SOC separates the cartilaginous epiphysis into two structures with different functional roles, i.e., the growth plate responsible for skeletal growth and the articular cartilage responsible for skeletal articulation (Fig. 1). The major differences between these two structures are as follows: the chondrocytes at the bottom of the growth plate undergo extensive hypertrophy and in the middle of this structure proliferate rapidly in the longitudinal direction. Eventually, in many species, including humans but not rodents, the growth plate fuses after puberty, in contrast to the articular cartilage, which is a more permanent structure [19].

## Two Sources of Endosteal Osteoblasts and the Fate of Hypertrophic Chondrocytes

Although one source of endosteal osteoblasts has been considered to be perichondrial cells, some of which migrate into the marrow cavity following invasion by blood vessels [12], in recent years, this viewpoint has been changing.

In fact, the fate of hypertrophic chondrocytes has long been debated. As early as 1890–1893, Van der Stricht and Brachet proposed that hypertrophic chondrocytes transdifferentiate into bone cells and occasional experimental studies; both ex vivo and involving morphological observations have supported this proposal [20, 21]. Further support was received as well from functional investigations where pieces of endochondral cartilage labeled with thymidine were transplanted under the skin, and the bone formed was found to contain thymidine-labeled osteoblasts [22]. However, contamination of such explants with perichondrial cells and/or osteoprogenitors cannot be fully excluded.

With the discovery of apoptosis, the concept that hypertrophic chondrocytes undergo terminal programmed cell death [23] has become the prominent dogma for almost 40 years. However, in 2014, this dogma was seriously challenged by three independent demonstrations that hypertrophic chondrocytes trans-differentiate into osteoblasts in vivo in a physiological setting [24–26]. All three of these research groups utilized fate mapping/lineage tracing but with different transgenic Cre strains of mice, including Col10-Cre and inducible Col10-CreERT2, Col2-CreERT, and aggrecan-CreERT2 strains.

Fate mapping and lineage tracing are powerful genetic approaches that allow for deciphering relationships between cell types. Fate mapping is the genetic labeling of a population of cells utilizing the Cre gene expressed under a tissue-specific promoter and a reporter gene, e.g., green fluorescent protein (GFP), activated by this Cre [27], and thereafter continues to be expressed by these cells and all their progeny. Lineage tracing employs the same principle but utilizes inducible Cre (usually CreERT [28, 29]), which allows the labeling of a specific population of cells to be done at any given time point and thereafter identifies all the descendant cells based on the reported expression.

Consequently, cross-breeding Col10-Cre and tdTomato reporter mice results in the expression of tdTomato by all hypertrophic chondrocytes, which are the only cells that express high levels of collagen type X [26]. However, not only were the tdTomato-expressing cells shown to be hypertrophic chondrocytes, as expected, but such expression was also observed unexpectedly in osteoblasts underlying the growth plate [26]. The experiments with mice carrying inducible genes encoding Col2-CreERT, Aggrecan-CreERT, and Col10-CreERT demonstrate clearly that many hypertrophic chondrocytes escape apoptosis and contribute to the generation of osteoblasts inside the bone marrow cavity [24–26].

Despite these highly convincing initial findings, it took several years before researchers began reevaluating the dogma. The major concerns were that Cre or tdTomato was being released from dead hypertrophic chondrocytes and being taken up by osteoblasts, or another concern is that the Col10 promoter is not sufficiently specific for hypertrophic chondrocytes but is also expressed by osteoblasts in low amounts sufficient for recombination, as is the case in zebrafish [30].

Today, ample evidence obtained from the clonal lineage tracing [16, 25], single-cell RNA sequencing (scRNAseq) [31••] and functional perturbations [32••] leaves no doubt that at least some hypertrophic chondrocytes undergo lineage extension to generate osteoblasts. In fact, certain estimates indicate that as much as 83% of the intramedullary osteoblasts originate from chondrocytes [31••], although multiple other reports propose that only approximately one-third to one-half of trabecular osteoblasts come from chondrocytes. The variation in these estimates reflects differences in the efficiencies of the Cre lines employed, the methods by which the osteoblasts are co-labeled, and the time-point in development when the analysis is performed [24–26, 31••, 32••]. Nonetheless, the clearly significant contribution of chondrocytes to the formation of osteoblasts provides an entirely new perspective on bone formation during endochondral ossification, as well as in connection with skeletal disease.

However, our understanding of the molecular mechanism(s) underlying the transition of chondrocytes to osteoblasts remains rudimental, although a few factors are already known to be involved. Both Osterix and Runx2, transcription factors that dictate osteogenesis, play essential roles in promoting this process [33, 34•], whereas Sox9, the master transcription factor involved in chondrogenesis, is inhibitory [35•]. Moreover, the transcription factors (Iroquois homeobox-containing transcription factors 3 and 5) IRX3 and IRX5 are cooperatively involved in the transition of hypertrophic chondrocytes toward the osteolineage, with their deficiency skewing this transition toward adipogenesis [36••].

In addition, inactivation of bone morphogenetic protein receptor 1A (BMPR1a) in chondrocytes completely blocks the transition of chondrocytes to osteoblasts and osteocytes, as well as leading to a lack of trabeculae under the growth plate [37]. This finding suggests that BMP signaling is also involved in the transdifferentiation of hypertrophic chondrocytes, but the impairment in chondrocyte hypertrophy in these same mice offers a potential alternative explanation [37].

Furthermore, activation of the canonical WNT β-catenin pathway in hypertrophic chondrocytes promotes the transition of hypertrophic chondrocytes toward osterix + -positive osteoprogenitors and extensive formation of trabecular bone, whereas inactivation of this pathway has the opposite effect [38]. Interestingly, β-catenin may regulate the lineage extension via IRX3/5 transcription factors [36••].

Yet, another factor recently discovered to be involved is parathyroid hormone (PTH), which promotes transdifferentiation of hypertrophic chondrocytes, while cleavage of its receptor (PTH1R) by MMP14 regulates the supply of chondrocyte-derived osteoblasts [32••]. scRNA-sequencing of Col10a1-Cre:tdTomato mice reveals that these osteoblasts are transcriptionally similar to and respond to PTH in the same manner as other osteoblasts, even as mice age [31••, 32••]. These latter observations indicate that osteoblasts that arise from two separate sources are morphologically, transcriptionally, and functionally similar.

Despite these initial insights into the mechanism(s) underlying this lineage extension, our understanding of the process is still at a very early stage.

## Transdifferentiation or Dedifferentiation?

In this context, it is of considerable importance to determine whether this lineage extension involves transdifferentiation (i.e., direction transition of a differentiated cell to another differentiated phenotype) or dedifferentiation followed by re-differentiation to another cell type. Initially, it was proposed that hypertrophic chondrocytes transdifferentiate into osteoblasts and/or osteocytes [26], whereas subsequent reports show that osterix-positive osteoprogenitors are also descendants of hypertrophic cells [35•], suggesting that transition occurs toward the entire osteolineage. At the same time, CXCL12-abundant reticular (CAR) cells and adipocytes are also generated from hypertrophic chondrocytes [17, 36••], which thereby indicates either multipotency of the generated intermediate cells or, alternatively, indicates that different hypertrophic chondrocytes transdifferentiate into different lineages.

In this connection, single-cell RNAseq of Col10-traced cells during their transition phase has revealed that these cells acquire several markers typically expressed by skeletal stem and progenitor cells (SSPCs), including LepR, CXCL12, PDGFRa, Grem1, and PDGFRb [31••, 39]. Lineage tracing of any one of these markers showed that the labeled cells were multipotent and each population was proposed to contain skeletal stem cells (SSCs) [18]. It is noteworthy that the contribution of descendants of hypertrophic chondrocytes to these subpopulations was considerable—for example, 74% of these descendants were LepR-positive [31••]. However, the contribution of chondrocytes to the osteolineage is most extensive during the period of growth early in life, whereas in mice LepR + cells begin contributing to the osteolineage at 2 months of age [39].

Altogether, the observations described above suggest that rather than transdifferentiating, hypertrophic chondrocytes first dedifferentiate and acquire stem cell-like properties. If so, it is fascinating that fully differentiated cells can give rise to multipotent progenitors under entirely physiological conditions.

**Fig. 2:**
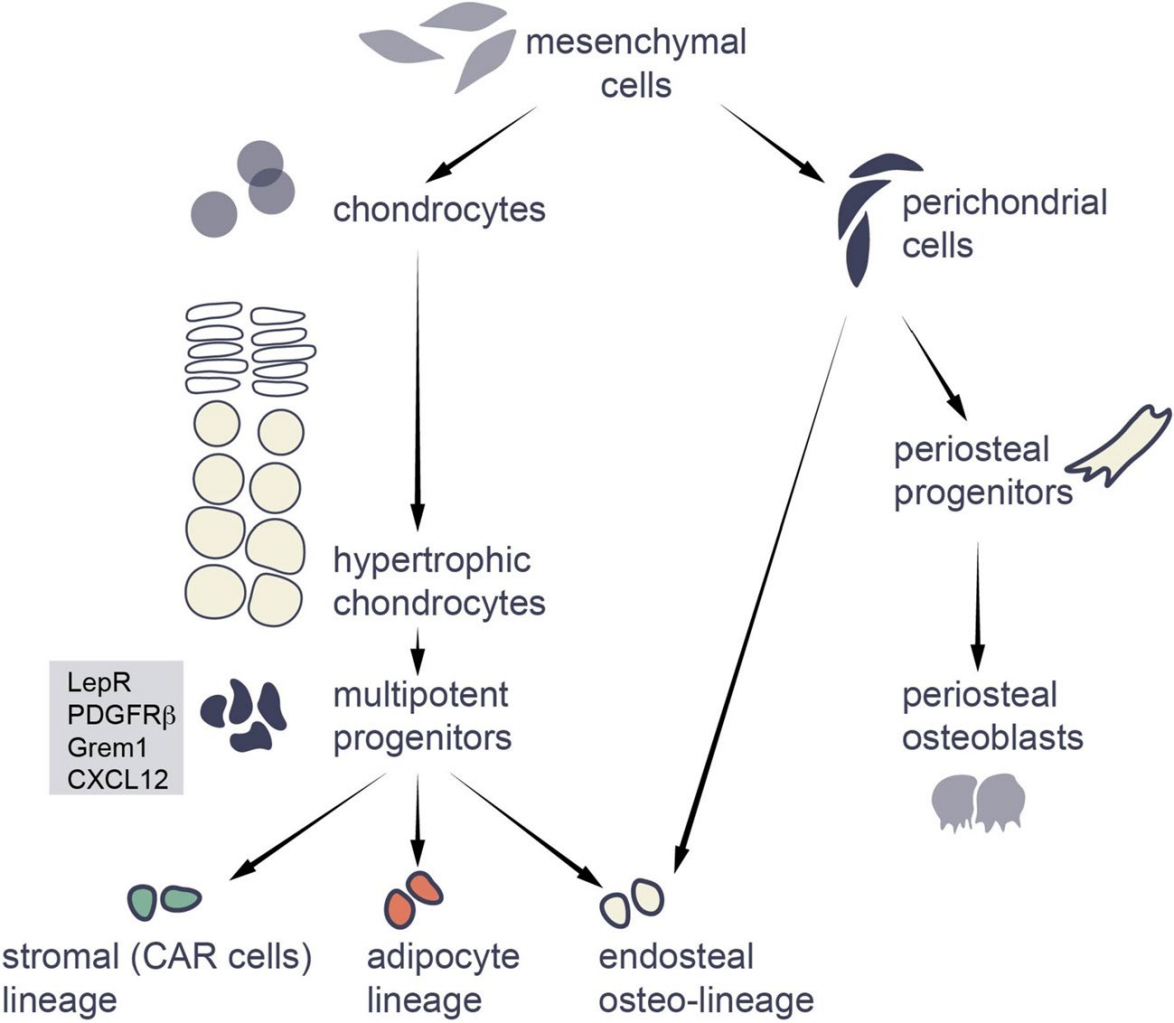
Mesenchymal cells become endosteal osteoblasts via two different routes of differentiation

This possibility becomes even more fascinating if we assume that the multipotent progenitors generated are long-lived skeletal stem cells (SSCs). This assumption is supported indirectly by the following observations: (i) the multipotency discussed above, (ii) the observation that descendants of Col10-positive chondrocytes labeled on postnatal day 9 in mice can be found in the bone marrow 16 months later [26, 32••], and (iii) the fact that descendants of hypertrophic chondrocytes begin to express LepR [31••], and LepR + cells are currently considered to be SSPCs containing mostly SSCs [39, 40] (Fig. 2).

From this perspective, it is of interest to reconsider certain studies in which mouse skeletal stem cells (mSSCs) were identified by FACS followed by transplantation under a renal capsule where the bone formation was evaluated. More specifically, CD45 − Ter119 − Tie2 − CD51 + Thy − 6C3 − CD105 − CD200 + mSSCs isolated from the area of the growth plate by FACS were found to generate bone that supported hematopoiesis [41]. Since the recent single-cell analysis has shown that descendants of Col10 + hypertrophic chondrocytes also express CD51 and CD200 [31••], it is plausible that transplantation of immediate descendants of hypertrophic chondrocytes can form this type of tissue. In support of this proposal, subcutaneous transplantation of iPS-derived hypertrophic chondrocytes leads to the formation of bone tissue that supports hematopoiesis, whereas similar transplantation of articular chondrocytes does not [42, 43•].

Thus, at least in theory, hypertrophic chondrocytes are a source of skeletal stem cells. The observations described above also indicate that chondrocyte hypertrophy is required for subsequent dedifferentiation. Since hypertrophy is the ultimate destiny of most of the chondrocytes in the growth plate, this entire structure may act as a continuous source of skeletal stem cells/progenitors during the period of growth.

**Fig. 3:**
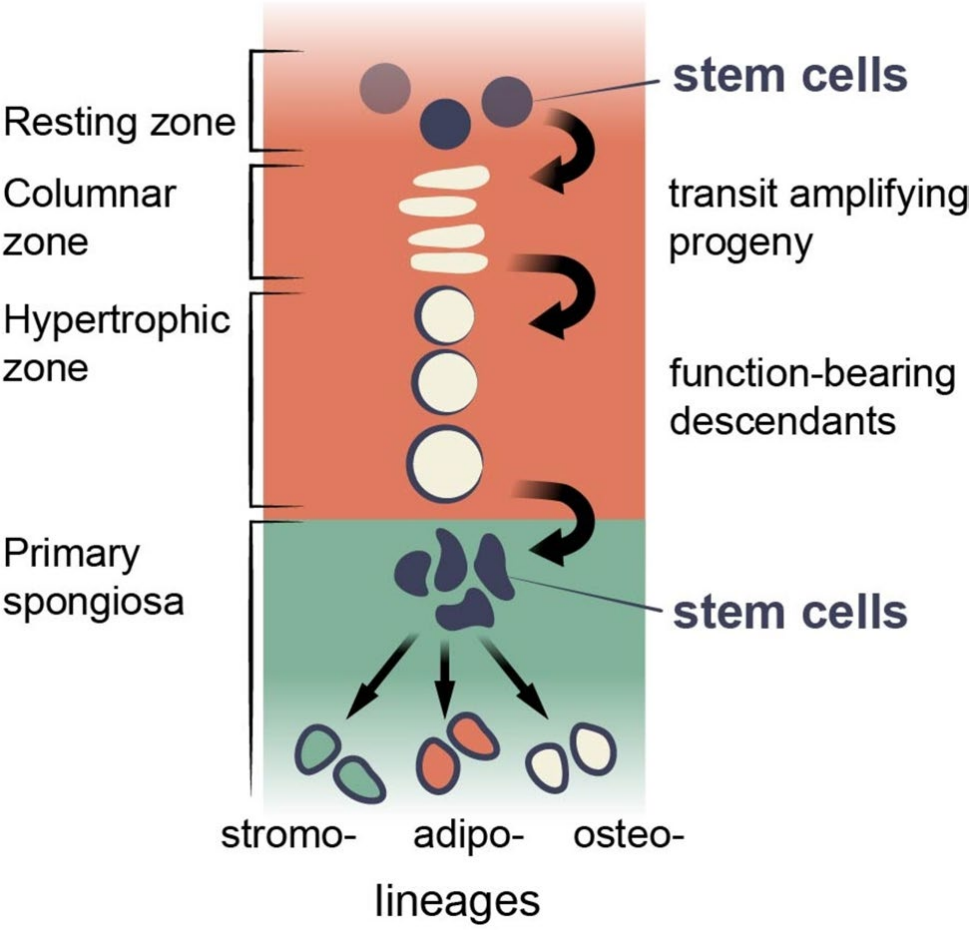
Schematic illustration of the proposed hierarchy of skeletal stem cells and their lineage transition in the postnatal bone

At the same time, the growth plate contains its own stem cells [17, 44] and stem cell niche [16, 45]. The most rigorously characterized are stem cells located in the resting zone of the postnatal growth plate and expressing PTHrP [17]. These stem cells are initially unipotent and under physiological conditions generate only chondrocytes, which then, after proliferating and undergoing hypertrophy, generate osteoblasts and CAR cells [17]. Pulse-chase experiments revealed that stem cells labeled at postnatal day 6 provide osteoblasts 12 months later, suggesting these PTHrP + stem cells are self-maintaining and simultaneously provide a continuous source of differentiated progeny throughout life [17]. Moreover, it is possible that the growth plate has two distinct subpopulations of stem cells since recently reported FoxA2 + cells, not much overlapping with the PTHrP + population, are located within the resting zone, capable of maintaining themselves simultaneously generating chondrocytes and subsequently osteoblasts for at least 9 months in mice [46]. Thus, altogether, present evidence is indicative of a paradoxical and highly uncommon physiological situation, where stem cells generate functionally differentiated progeny, i.e., hypertrophic chondrocytes, which subsequently dedifferentiate into another type of stem cell capable of generating other types of differentiated cells performing other functions (Fig. 3).

## Paradox or Plasticity?

The discovery of cell plasticity, i.e., the ability of a differentiated cell to dedifferentiate into a more primitive un- or partially differentiated stage and thereafter give rise to a different lineage, was awarded the Nobel Prize in 2012. This process occurs relatively often during embryonic development, with the most striking examples being epithelial-to-mesenchymal and mesenchymal-to-epithelial transitions such as those that take place during the formation of the neural crest cells from neuroectoderm and epithelization of the mesoderm during somitogenesis, respectively. In postnatal life, cell plasticity is also observed, but generally in connection with cell loss, injury, inflammation, and other artificial or pathological perturbations (reviewed in [47]).

Thus, the growth plate constitutes the first case where such plasticity is clearly employed to accomplish entirely normal physiological functions (Fig. 2). Furthermore, the mesenchymal cells of the same condensation which escape chondrogenic differentiation (i.e., remain at the edges of chondrogenic condensation and form the perichondrium) later give rise to cells of the osteolineage within the marrow cavity without such plasticity [12, 48]. Thus, osteoblasts in the marrow cavity are generated via two different mechanisms, one direct and the other involving cells of the osteolineage obtained via a chondrocyte intermediate (Fig. 2).

It is noteworthy that the osterix + progenitors which arise early during development are relatively multipotent, generating not only cells of the osteoblast lineage but also the stromal population in the bone marrow [48]. Furthermore, embryonic osterix + cells labeled during the formation of the primary ossification centers give rise to transient populations of stromal and osteolineage cells, whereas those labeled during the perinatal period give rise to long-lived populations of these same cells [48]. In light of the fact that osterix + cells are generated from hypertrophic chondrocytes [38], it is worth exploring the possibility that at least a substantial part of these long-lived populations is, in fact, derived from descendants of hypertrophic chondrocytes.

## The Plasticity of Articular Chondrocytes

Is this remarkable plasticity a property of hypertrophic chondrocytes only or is it displayed by other chondrocytes as well? Unlike epiphyseal cartilage, subcutaneous transplantation of articular cartilage does not lead to the formation of the bone [42]. Since articular cartilage is a permanent structure, supposed to remain throughout life, with little, if any capacity for renewal [49], adult articular chondrocytes would not be expected to undergo dedifferentiation under normal physiological conditions.

However, under pathological conditions such as osteoarthritis, articular chondrocytes can undergo hypertrophy and form bony osteophytes. A recent study found very little contribution of Col2-traced cells to the bone tissue of osteophytes, although a contribution by Sox9-traced cells was observed [50]. In cases of severe osteoarthritis, cells whose morphology resembled that of fibroblasts and which exhibited multipotency and an extensive ability to migrate were observed in the human articular cartilage [51]. However, it is impossible to determine whether these cells originated from chondrocytes or had migrated from the underlying bone marrow without performing lineage tracing.

As far as we are aware, no linear tracing specifically of articular chondrocytes has yet been reported, but certain sporadic observations are worth mentioning in this context. When chondrocytes in neonatal Co121-CreERT mice are traced, labeled cells can later be observed in the bone marrow of the SOC, immediately beneath the articular cartilage [49], suggesting that they have transdifferentiated. However, Col2-CreERT can label the perichondrium as well, which may also contribute to cells of the osteolineage within the SOC [13]. Slightly more convincing are the findings that upon lineage tracing with Prg4-CreERT2 animals, the superficial labeled cells first become articular cartilage and thereafter appear in the underlying bone and marrow of the SOC [52]. However, such transition occurs only if the cells are traced from embryonic day E18.5, but not when traced from birth ([49] and our own unpublished observations).

At the same time, the plasticity of articular chondrocytes in vitro is very well established [53], since in culture these cells readily lose their phenotype and alter their epigenetic and transcriptomic profiles [54]. Chondrocytes labeled genetically in vivo can differentiate into osteoblasts and adipocytes ex vivo, largely excluding the possibility of contamination by other types of cells [55•]. Consequently, it appears likely that under pathological conditions and ex vivo, articular chondrocytes exhibit relatively extensive plasticity and thus may also dedifferentiate during the neonatal period. While stabilization of their phenotype throughout adult life is likely intrinsic, it can also be attributed to the specific microenvironment in the synovial joint, since growth plate chondrocytes transplanted into this microenvironment acquire the characteristics of articular chondrocytes [56•].

## Clinical Considerations

It is not yet known whether hypertrophic chondrocytes give rise to cells of other stromal lineages in the bone marrow of humans, even though transdifferentiation of iPS-derived human hypertrophic chondrocytes upon implantation into mice [43•] suggests that this is the case. The presence of skeletal stem cells in the vicinity of the fetal human growth plate and the ability of these cells to form bone which supports hematopoiesis upon transplantation beneath the renal capsule [57] raise the possibility that these cells are dedifferentiated hypertrophic chondrocytes, in analogy to the situation in mice described above [41]. However, these observations are indirect, and more definitive evidence is required.

It is important to emphasize that in contrast to rodents, the growth plates in humans disappear through a process referred to as fusion or closure during late puberty [19] via a mechanism not involving apoptotic cell death [58]. After this fusion, bone homeostasis continues with no contribution from the growth plate. Thus, even if trans- and/or dedifferentiation of hypertrophic chondrocytes does occur in humans, the contribution of such a process to bone formation is limited to the period of linear growth, unless, of course, even after fusion dedifferentiated progenies remain in the bone as long-lived skeletal stem and progenitor cells.

Until these questions have been answered, murine osteoporotic models must be applied to human pathophysiology with great caution, particularly models concerning the regulation of trabecular bone in the proximity of the growth plate. Indeed, murine growth plates continue to produce hypertrophic cells until 4–12 months of age, depending on the strain, thereby providing an additional supply of osteoblasts which is absent in humans.

## Evolutionary Considerations

Is there any potential evolutionary explanation for the paradoxical transitions of cell fate described above?

It appears likely that chondrogenesis and osteogenesis developed independently during evolution. Cartilage is a very ancient tissue the development of which is regulated by SoxD (mammalian orthologs Sox 5/6/13), SoxE (mammalian orthologs Sox8/9/10), and ColA (mammalian orthologs Col2a1, Col1a1). This tissue appeared prior to the splitting of the animal kingdom into protostomes and deuterostomes, i.e., 670 million years ago, and cartilaginous structures are present in such diverse creatures as mammals, squids, and horseshow crabs [59–61].

The family of calcium-binding phosphoproteins, including enamel and dentin proteins, sialoproteins, osteopontin, and casein, began to evolve from SPARC-like gene duplications approximately 600 million years ago [62, 63]. At the same time, phosphate-based skeletal tissues, i.e., dentin, enamel, and bone, first appeared during evolution as tooth-like structures called odontodes in the skin of jawless eel-like chordates known as conodonts [64]. Subsequently, scale-like dermal skeletal structures resembling these odontodes rapidly evolved into dermal bone plates that form extensive exoskeletons in ostracoderms (jawless fish) and placoderms (jawed fish) [65] approximately 470–430 million years ago. Thus, the cartilaginous endoskeleton and bony dermal exoskeleton are likely to have evolved independently.

In modern tetrapods, the formation of the cranial skeleton is intramembranous, in a manner similar to the corresponding process in armored fish such as ostracoderms and placoderms. At the same time, most of our skeletal structures are formed via endochondral ossification, i.e., based on a cartilage template. So, when, why, and how does this occur and what role may transdifferentiation play in this context?

Ossification of the endoskeleton appeared first in early *Gnathostomata* (jawed animals) in the form of a perichondral ossification [66]. This process involves the formation of bone on the surface of cartilage produced by the adjacent mesenchymal cells, usually as a tube-like structure around the midsection of the cartilage, which probably provides additional stiffness to the cartilaginous elements. At the same time, periosteal ossification also occurs in many extant animals, including the formation of the jawbone of mammals around Meckel cartilage and in most bones of teleost fish. In this case, bone is formed without the intermediate involvement of chondrocytes, building around a cartilage anlage and thereby armoring the initially cartilaginous endoskeleton. The cartilage anlage can remain inside its bony shield for a long time, gradually degenerating.

Thus, perichondral ossification resembles dermal ossification, except that the former takes place along cartilaginous elements. It seems plausible that the genetic program that evolved for intramembranous ossification was later adopted for perichondral ossification in order to reinforce the endoskeleton with more rigid structures while retaining the growth rate and potential for locomotion.

Endochondral bone formation, in connection with which a cartilage template is replaced by the bone, appeared more recently during evolution than intramembranous and perichondral ossification. Samples of stem sarcopterygians (lobe-finned fish), 380 million years old, exhibit signs of such bone formation [67], and all terrestrial animals that evolved from these fish, including humans, also demonstrate endochondral bone formation. Since certain teleost fish also show endochondral ossification and growth plates, it is possible that endochondral bone formation evolved in vertebrates prior to their split into sarcopterygians and actinopterygians [66] 400 million years ago.

It is generally considered that endochondral bone formation gives rise to both bone collars (i.e., cortical bone) and spongy or trabecular bone. Recent lineage tracing in mice revealed that bone collars are formed directly from the perichondrium, without a cartilage intermediate [68] and, therefore, are formed by perichondral ossification, which appeared prior to endochondral ossification during evolution. In the case of mammals and reptiles, the cortical bone collars grow at the periphery of the growth plates, while the trabecular bone is formed immediately beneath this plate, with both processes being, in general, tightly coupled. However, in amphibians, this coupling is not as tight; for instance, in *Urodela* (e.g., newts and salamanders), periosteal ossification often lags behind the bone growth, whereas in *Anura* (e.g., frogs and toads), periosteal ossification is more rapid than endochondral, so that a large proportion of the cartilage remains within bony collars [69–71]. Moreover, in connection with limb regeneration in newts and salamanders, these two processes are largely uncoupled, with periosteal ossification taking place after the cartilage template has regrown almost to full size [72]. Interestingly, uncoupling of periosteal and endochondral ossification can also be observed in humans under certain pathological conditions, e.g., metatrophic dysplasia [73].

Thus, both evolutionarily and developmentally, the formation of bone collars and of trabeculae can be viewed as two separate processes. From this perspective, hypertrophic chondrocytes function specifically as a source of cells of the osteolineage for trabecular bone formation. Trabeculae, which improve the strength of bony collars, are particularly beneficial for the weight-bearing demands placed on terrestrial animals.

During endochondral bone growth, longitudinal trabeculae are formed on the calcified cartilage template. An additional source of cells for use in the formation of trabeculae would be clearly advantageous in evolutionary terms. Interestingly, in extant *Teleost* fish and *Urodele* amphibians, trabeculation is relatively undeveloped, with the bone marrow cavity being filled predominantly with adipose cells [74–76]. Whether these adipocytes are descendants of hypertrophic chondrocytes remains unknown, although there is one report demonstrating such a transition in zebrafish [76]. On the other hand, longitudinally oriented trabeculae were reported in stem lobe-finned fish [67], suggesting that extant *Urodele* may have lost the ability to form these structures during evolution.

In conclusion, at present, it is impossible to say whether the hypertrophic chondrocytes of teleost and mammals acquired plasticity independently, whether initially hypertrophic cells transitioned into adipose tissue, and/or whether osteopotential was acquired later in the evolution. Nevertheless, chondrocyte hypertrophy and their subsequent transition toward osteolineage coupled with the formation of trabeculae have a clear evolutionary advantage, particularly for weight-bearing terrestrial vertebrates.
